# Recurrent Spontaneous Intracranial Hypotension (SIH) and the Durability of Repeat Epidural Blood Patch (EBP)

**DOI:** 10.7759/cureus.41457

**Published:** 2023-07-06

**Authors:** Ali Hazama, Fakhri Awawdeh, Alexander Braley, John Loree, Amar Swarnkar, Lawrence S. Chin, Satish Krishnamurthy

**Affiliations:** 1 Neurosurgery, The State University of New York Upstate Medical University, Syracuse, USA; 2 Neurological Surgery, Lake Erie College of Osteopathic Medicine, Elmira, USA; 3 General Surgery, Temple University Hospital, Philadelphia, USA; 4 Neuroradiology, The State University of New York Upstate Medical University, Syracuse, USA

**Keywords:** orthostatic headache, spontaneous cerebrospinal fluid leak, subdural hematoma, dural leak, blood patch, spontaneous intracranial hypotension (sih)

## Abstract

Objective: Spontaneous intracranial hypotension (SIH) remains a rare and difficult clinical entity to diagnose and treat. Epidural blood patch (EBP) of the dural sac is the mainstay definitive treatment for refractory cases and has mixed efficacy. We sought to evaluate the recent efficacy and outcomes of EBP for SIH at our institution.

Methods: Twenty-three patients (14 women, 9 men, mean age 49) were seen and treated for SIH between Summer 2009 and Spring 2018 at the same institution. All patients underwent brain MRI with and without gadolinium contrast and T2-weighted spine MRI. Targeted EBP was placed one or two vertebral levels below areas of suspected leak, while the patient was positioned in the lateral decubitus position. Patients were seen in the outpatient setting within a week following initial EBP and repeat EBP was offered to patients with persistent symptoms. Patients were followed if symptoms persisted or for 6 months following clinical relief of symptoms.

Results: 22/23 (95.7%) patients presented with complaints of orthostatic headache, and 3 (13%) patients presented with altered mental status (AMS) or focal neurologic deficit. Brain MRI demonstrated pachymeningeal enhancement in 16/23 (69.6%) patients, and 5/23 (21.7%) patients had a subdural hematoma (SDH) present. Dural leaks were successfully identified in 18/23 (78.3%) patients. 12/23 (52.2%) patients had symptomatic relief with initial EBP, and 5/23 (21.7%) patients received further EBPs for persistent disease with all achieving relief after repeat EBP. 5/12 (41.7%) of patients had recurrent symptoms after initial relief with EBP, and 4/5 (80%) were successfully treated with a second EBP. The mean initial EBP volume and number of EBPs per patient were 21.7 mL (median 20 mL, 7-40 mL) and 3.54 (median 1, 1-13) respectively. There was one complication from initial EBP (cervical dural tear requiring operative closure) treated with open surgical management successfully. In total, 18/23 (78.2%) patients are currently asymptomatic with regard to their SIH. The mean follow-up in this cohort was 2.6 years (median 1.8 years, 1.8 months-9.27 years).

Conclusions: EBP is a viable and effective option for the treatment of recurrent SIH caused by cerebrospinal fluid (CSF) leaks.

## Introduction

Spontaneous intracranial hypotension (SIH) is a syndrome characterized by orthostatic headache and low cerebrospinal fluid (CSF) pressure that is associated with a spectrum of other neurologic and gait manifestations ranging from mild to severe [1]. Classic imaging findings associated with this diagnosis are diffuse pachymeningeal enhancement on brain MRI, venous engorgement, pontine flattening, and low-lying cerebellar tonsils [2-4]. The most severe presentation of this syndrome manifesting as subdural hematoma (SDH) is present on imaging with or without altered mental status (AMS) and focal neurologic deficit [5-8]. Generally, this condition is due to CSF leaks around the spinal meningeal diverticula, microscopic dural tears, or escape around nerve roots exiting the spinal column. However, the exact etiology and pathophysiology of these lesions are difficult to determine [7-11].

Primary treatment of SIH is often conservative, with bed rest, aggressive hydration, and caffeine. For patients with symptoms not responding to these conservative therapies, invasive therapies may be required. These invasive modalities include epidural blood patch (EBP) vs open surgery and direct dural repair [12-13]. For illustration, an open surgical repair of a spinal spontaneous CSF leak would involve a skin incision, muscle dissection, laminectomy/laminoplasty, and finally ligamentum flavum and epidural fat removal in order to expose the dura in order to repair a leak with sutures, bolsters, or sealants. This typically requires a short hospitalization and is associated with surgical risks such as blood loss, neural injury, infection, wound healing issues, etc. Alternatively, EBP can be done outpatient and involves a small needle stick. Given the stark difference in morbidity associated with these treatments, EBP is the preferred therapy for many practitioners [1, 5, 7-12].

The EBP, however, poses several challenges to implementation. First, the rate of successful MRI localization for leak sites is low, reported between 20% and 90%, varying significantly between institutions and MRI findings are not usually predictive of treatment success [2-3, 6]. In addition, EBP is reported to have a low initial success rate, reported between 50% and 70% [5-6].

Spontaneous intracranial hypotension remains a rare disease, with an incidence estimated at 5 per 100 000 persons per year, and there remains insufficient data on the efficacy of EBP in the treatment of this clinical entity [13]. There is no standard protocol in the literature for when and how to give EBP, and even less guidance on the management of recurrent SIH. As such, we share recent experiences with EBP at our institution.

## Materials and methods

Institutional IRB approval under exemption was obtained by the Upstate Medical University institutional review board. Between June 2009 and February 2018. During this time 23 patients with SIH treated with EBP at our institution were identified. SIH was diagnosed under the following circumstances, clinical symptoms of orthostatic headache with or without other neurologic symptoms, evidence of CSF leakage on MRI or CT cisternography suggestive of intracranial hypotension (pachymeningeal enhancement, cerebellar tonsil descent with or without SDH, venous engorgement, paraspinal extravasation of CSF, no recent history of injury or procedure which may have disrupted the dura, and not attributable to other disorders). It is important to note that at least one of these three criteria must be met for inclusion.

Patients were evaluated either by Neurology or Neurosurgery and were treated primarily inpatient until the first EBP. Initial treatment varied based on admitting service, however, most patients were conservatively treated with bed rest, hydration, and caffeine prior to the scheduling of EBP. All patients with SIH received an MRI of the brain and spine with gadolinium contrast prior to EBP. The decision to proceed to EBP was based on the clinical scenario including ruling out all other possible factors, of orthostatic headache, the presence of at least one of the following: low opening pressure (≤60 mmH2O), cranial MRI changes of intracranial hypotension (e.g., brain sagging or pachymeningeal enhancement); no recent history of dural puncture; not attributable to another disorder with patients scheduled for EBP with interventional radiology within the hospital. CT cisternography was carried out prior to the procedure, and EBP placement was based on the results of imaging studies.

The EBP procedures were carried out with the patient awake in the lateral decubitus position with 1% lidocaine without epinephrine local anesthetic. All EBP procedures were performed under strict aseptic conditions. 18G spinal needles were used for EBP and were inserted under X-ray guidance. Slow injection of autologous venous blood was carried out to a maximum of 40 mL, stopping earlier if the patient complained of significant pain, headache, numbness, or significant resistance was encountered in the syringe. Patients were maintained in a prone position for 30 min following the completion of the EBP. Strict bed rest was recommended until clinical evaluation at morning rounds the next day. In patients who had no response to EBP or had a relapse following EBP, repeat EBP was offered after a minimum of one week.

For descriptive analysis, categorical data were expressed as numbers and percentages. Treatment success was considered the clinical improvement of symptoms such that the patient was satisfied with their therapy. Statistical testing was carried out using the Fisher Exact test with categorical groupings and two-tailed t-testing for continuous data. Significance was tested at a level of 0.05 with all significance-generating tests two-sided.

## Results

Patient demographics and presentation information are listed below (Table 1). There were 23 patients identified during the review seen between June 2009 and February 2018. All patients were diagnosed with SIH without previous interventions or traumas causing dural injury. One patient had a stable ~1 cm right temporal lobe mass on MRI which had been stable for 10 years prior to diagnosis of SIH. All patients had an MRI brain and spine with contrast and received at least one EBP at our institution. Twelve (52.2%) patients had symptomatic relief with initial EBP. Ten (43.4%) patients had repeat EBP at this institution. Overall, 18 patients (78.2%) patients had symptomatic relief with EBP as of this article. Core results are summarized below as well (Table 2). 

**Table 1:** Patient characteristics. This table is dedicated toward specific characteristics in our patients whether female or male, including associated symptoms seen in these patients, and coagulation status. The goal is to evaluate the differences and similarities in presentation between males and females, and ultimately how this impacted their treatment course.

Characteristic	N	%
Age, Years, Median (Range)	51 (19-74)	-
Sex		
Male	9	39.1
Female	14	60.9
Body Mass Index (Range)		
	27.0 (16.7-42.6)	-
Presenting Symptoms		
Headache	22	95.7
Nausea	11	47.8
Photophobia	6	26.1
Altered Mental Status	2	8.7
Neurologic Deficit	1	4.3
On Anticoagulation		
Yes	2	8.7
No	21	91.3

**Table 2:** Core results. This table highlights the overall results and findings within our study. This shows the localization of the EBP whether it was localized to a specific area that was noted or indirectly to the best of abilities. Furthermore, it presents with statistics on the need for a repeat EBP, how long the average patient follow-up was, and the overall relief of symptoms. EBP, epidural blood patch

Characteristic	N	%
Number of Patients	23	
EBP Localization		
Direct	12	52.2
Indirect	11	47.8
Mean Volume of Initial EBP (Range)	21.7 (7-40)	
Symptomatic Relief at First Followup		
Not Relieved	11	47.8
Relieved	12	52.2
Mean Follow-up Time (median, range)	2.6 years (1.8 yrs, 2 months-6.9 yrs)	
Complications	1 (cervical dural injury)	4.3
Repeat EBP		
Required	10	43.4
Not Required	13	56.6
Mean # Repeat EBP (median, Range)	1.9 (4.4, 1-12)	
Final Relief	18	78.2

Twenty-two patients presented with orthostatic headache, and one patient had a primary complaint of orthostatic neck and shoulder pain. Other clinical manifestations included lightheadedness, neck pain, nausea, vomiting, vertigo, tinnitus, shoulder/arm pain, photophobia, or phonophobia. Patients were symptomatic prior to presentation for a median of 35 days (0 days-5.75 years). Presentation varied substantially between patients, some acute, and some with insidious worsening of symptoms.

Twenty patients had findings suggestive of SIH on MRI, and 18 patients had specific leak sites localized via CT cisternography. Details of imaging results are reported in the table below (Table 3). Moreover, medicinal pre-EBP treatment in the inpatient setting was carried out in 11 (47.8%) patients, with the most common agents used being caffeine, non-steroidal anti-inflammatory medications, opioids, tricyclic antidepressants, magnesium, and anti-histaminergic medications.

**Table 3:** Imaging results. This table exhibits MRI vs CT findings and the different sites that were appreciated on imaging.

Characteristic	N	%
MRI Findings Present	20	87.0
Pachymeningial enhancement	16	69.6
Cerebellar Tonsillar Descent	9	39.1
Subdural Hematoma	5	21.7
CT Cisternography Findings Present	18	78.3
Cervical	1	4.3
Thoracic	12	52.2
Lumbar	12	52.2
Multiple Sites	10	43.5

**Figure 1:**
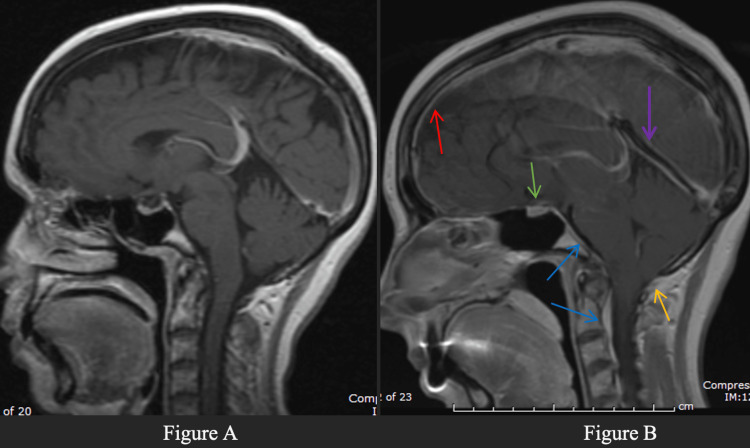
Normal MRI vs MRI with appreciated SIH. A: This is a normal MRI brain scan (left, sagittal postcontrast T1W pulse sequence) in same patient done 7 years ago. B: Typical findings of intracranial hypotension seen in current study shown above. This MRI was done 7 years from the previous scan after patient presented with multiple symptoms of headache, nausea, and left sided worsening weakness. Diffuse dural thickening and enhancement (red arrow). Venous engorgement along clivus and epidural veins, and a flattened pons (blue arrows). Lower position of cerebellar tonsils (yellow arrow). Pituitary stalk is curving posteriorly due to brain stem descent (green arrow). Congestion of the straight sinus (purple arrow). SIH, spontaneous intracranial hypotension

**Figure 2:**
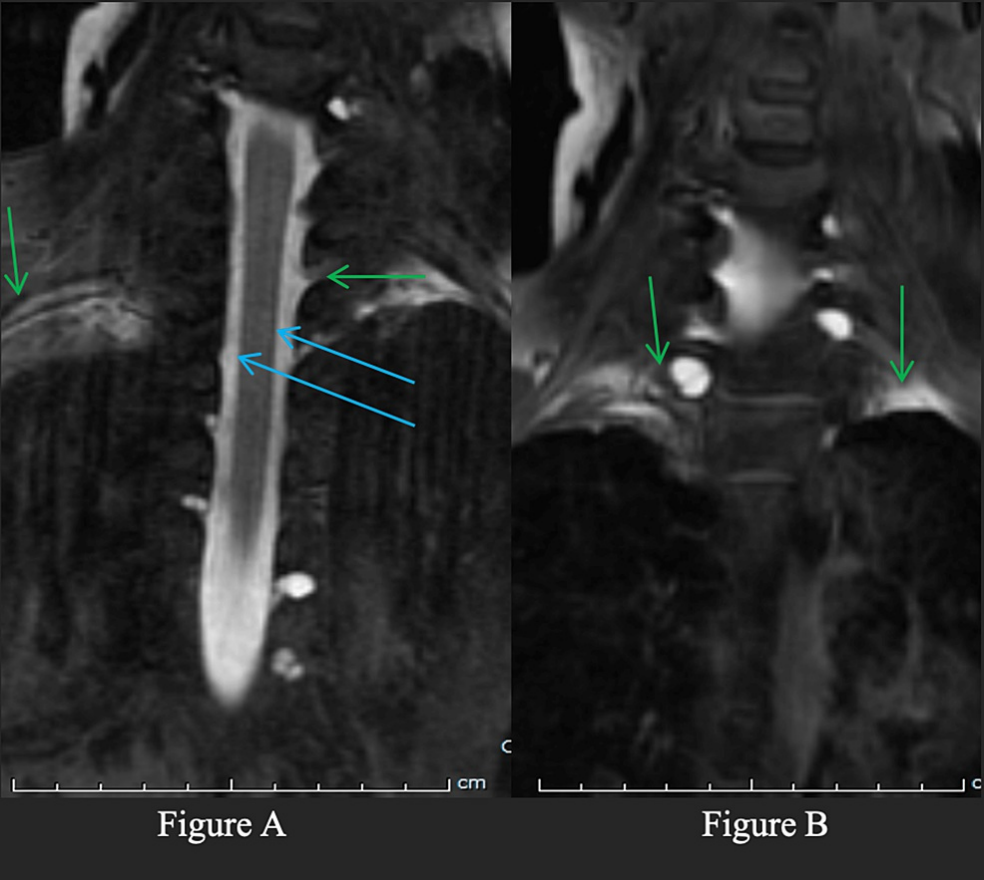
MR T1 weighted myelogram. MR T1 weighted myelogram with intrathecal gadolinium demonstrating bilateral CSF leaks is present in cervicothoracic region (green arrows). A small diverticulum is demonstrated by the blue arrows. A: This is a coronal view of the cervicothoracic region. There is a small diverticulum which is shown in the blue arrows. Furthermore, the green arrows point to the bilateral CSF leaks present in the imaging. B: This is another coronal view of the cervicothoracic region which illustrates the bilateral CSF leaks as shown by the green arrows. CSF, cerebrospinal fluid

**Figure 3:**
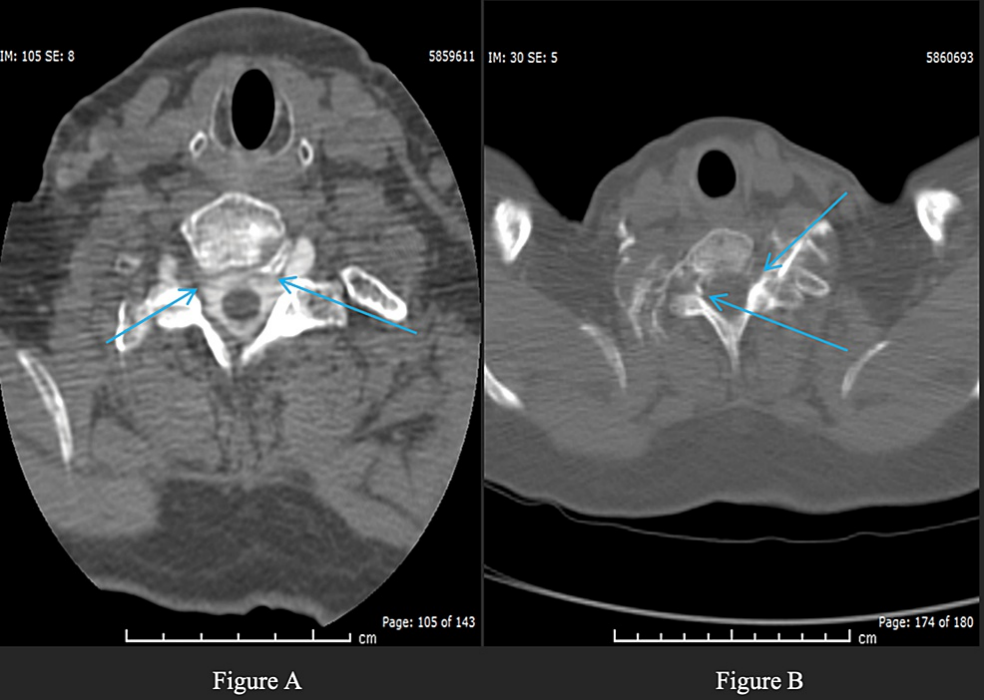
CT myelogram showing bilateral nerve root diverticula. CT myelogram showing bilateral nerve root diverticula without identifiable contrast leak. After corresponding to MR myelographic findings of CSF leak of Figure 2, bilateral foraminal epidural blood patches were performed. Finally contrast MR myelogram performed demonstrated CSF leak in cervicothoracic region. A: This illustrates an axial slice of the CT imaging that points to the bilateral nerve root diverticula, but unfortunately does not show an identifiable contrast leak for accurate administration of the EBP. B: This also illustrates a different axial which shows bilateral nerve root diverticula, but unfortunately does not show an identifiable leak. EBP, epidural blood patch

Direct targeting for EBP for the suspected leak was achieved in 12/23 (52%) patients, an example of pathology that would signify direct targeting by C MRI is shown in Figures 1-2. Initially, a CT myelogram was obtained but did not reveal any significant contrast leak as shown below (Figure 3). The mean initial EBP volume was 21.7 mL (median 20 mL, range 7-40 mL). One patient had a complication from the EBP itself (worsening cervical CSF leak causing AMS). The mean duration of relief with initial EBP was 2.2 years (range 24 days-6 years) with five (21.7%) patients experiencing recurrent symptoms. T-testing did not show an absolute or significant difference in relief duration according to initial volume injected, sex, age, BMI, localization, or presence of SDH (p >0.05).

The initial volume injected, sex, age, BMI, localization, EBP targeting, and presence of SDH were not predictive of initial EBP success. There was an absolute trend of men having better initial and sustained relief with EBP (77.8% and 55.6% vs 35.7% and 14.3%, p=0.09 and p=0.06). Additionally, there was an absolute trend in patients with BMI over the mean of this population having improved initial relief (66.7% vs. 36.3%, p=0.22). Finally, there was an absolute trend of directed EBP having improved sustained relief over indirect EBP (42% vs 18%).

Of the 10 patients with repeat EBP, five (50%) had recurrent symptoms, and all had eventual relief with repeat EBP. The mean number of additional EBP required to achieve remission was 2.54 (range 1-12). Two patients (8.7%) proceeded to open surgical management after initial EBP failure, both are currently asymptomatic. Of the five patients with recurrent symptoms, four (80%) were relieved with repeat EBP. In total, 18 (78.2%) patients achieved symptomatic relief as of this study (seven with initial EBP without recurrence, four with recurrence of symptoms, five after repeat EBP for persistent symptoms, and two with surgery). The initial volume injected, age, sex, BMI, localization, and presence of SDH were not predictive of long-term treatment success or the need for additional EBPs.

In patients with SDH present, initial EBP was successful in relieving symptoms and reducing SDH size in 80% of patients. Of those who received an initial EBP, 40% experienced durable relief with a single EBP. However, of the four patients who had resolution of their SDH with initial EBP on MRI, none had a recurrence of their SDH.

## Discussion

Spontaneous intracranial hypotension is a clinical syndrome attributed to small tears in the dura mater due to friction against osteophytes or trauma to the central column [1, 5]. The incidence of this problem is approximately 5/100,000 persons per year, with a higher prevalence in middle-aged women [13]. Similar to the symptoms listed by Mokri and reported by others, the most common manifestation of this syndrome is orthostatic headache [1, 4-9]. Over ninety percent of patients in the literature have a core symptom of orthostatic headache, which is corroborated in this study.

Like other studies, conservative treatment does not appear to have a high success rate [1-2, 5-7]. This finding was corroborated in this study, with a success rate of 27.3%, achieved only with caffeine-containing regimens. However, there are few studies published that focus on conservative treatments, and there have been no properly controlled randomized trials on this topic. As such, it is reasonable to expect that minimally invasive treatments, such as EBP will continue to be routinely used in the management of SIH.

The EBP however, is not a perfect intervention. Significantly, there is a low rate of initial EBP success, with a rate of 52% in this study, and rates between 50% and 90% reported in the literature [1-2, 4-6, 9]. In addition, although localization of CSF was achieved in 78% of patients, that leaves a sizeable portion of patients who require large volume indirect EBP, which is less effective than targeted EBP [5-6]. In this study, while not statistically significant, patients with indirect EBP recurred at a higher rate after initial EBP (82% indirect vs 58% direct). In addition, there is no clear means to distinguish which patients achieve symptomatic relief with initial EBP, with the possible exception of men performing better. This result, however, was not statistically significant in this series and is not reported as such elsewhere [5-6]. Furthermore, differences in success in such pathology can be due to the technique and expertise of the performing physician. Furthermore, advances in MRI have significantly improved the ability to detect CSF leaks with increased specificity. 

Perhaps most surprisingly, however, although initial EBP has a somewhat poor rate of success, repeat EBP tends to be highly effective. Of the 10 patients who received repeat EBP at our institution, nine (90%) experienced durable relief with repeat EBP. This effect has been reported elsewhere in the literature and substantially improves the clinical utility of EBP [5-6, 9-10]. Additionally, strict bed rest of about 24-36 h after EBP and appropriate interval, between 24 and 48 h, between EBP may improve the likelihood of success.

Although this patient series failed to demonstrate demographic or presenting characteristics that are predictive of relief success, there were absolute trends in male patients and patients with greater BMI gaining better initial relief from EBP although these findings did not reach statistical significance in the difference in volume injected during EBP by sex and BMI (t-test p=0.79 and p=0.41, respectively). The reasons for these trends are unclear, and they may be related to social pressures, differences in pain tolerances, anatomic variation, or ease of procedure between these groups. An example of these social pressures may be due to individuals with greater BMI believing that they must have improved outcomes or it may be attributed to their BMI. 

In addition, it appears that EBP is an effective means to reduce SDH caused by SIH. In our cohort, 80% of patients with SDH had resolution of the hematoma on imaging following initial EBP. Rates in the literature are similar, with reported response rates between 70% and 100% without surgery [4-6, 9]. The volume of blood injected into the epidural space helps to increase CSF pressure while decreasing the downward traction on the brain and meningeal structures, thus alleviating symptoms related to the condition. This effect is also seen with more acute presentations of SIH, including altered mental status and coma. As such, more aggressive management of this serious neurologic condition with EBP is likely preferred in this context.

Lastly, it would be a disservice not to mention the limitations of this study. The first is the length of the study spanning about 10 years which brings on the question of improved imaging and the ability to detect these leaks. This has significantly improved within the last 10-15 years based on improved imaging modalities and increased specificity. The evaluation of echo images greatly improved the detail of the osseous anatomy in the skull base which can lead to earlier detection and furthermore improved outcomes. Another limitation to be highlighted is the treatment of indirect EBPs. While this was not the target of our research, there should be more focus on how to treat and better localize these leaks in a systematic way.

## Conclusions

An epidural blood patch is traditionally used in the context of SIH to treat cases refractory to conservative management. This is a well-tolerated procedure that is minimally invasive, compared to open surgical dural repair. Based on the data presented herein and, in the literature, EBP appears to be a viable method in all clinical contexts of SIH. Perhaps most importantly, failure of the initial EBP is not adequate grounds to discontinue treatment with EBP, as a large portion of patients require multiple EBPs with significant subsequent success in clinical and radiologic signs and symptoms of SIH. Further investigation into the stratification of a patient’s need for repeat EBP is warranted, as it could tailor treatment and consultation of patients with SIH.
